# Obstructive Sleep Apnea and the Risk of Atopic Dermatitis: A Population-Based Case Control Study

**DOI:** 10.1371/journal.pone.0089656

**Published:** 2014-02-25

**Authors:** Kai-Jen Tien, Chien-Wen Chou, Shang-Yu Lee, Nai-Cheng Yeh, Chwen-Yi Yang, Feng-Chieh Yen, Jhi-Joung Wang, Shih-Feng Weng

**Affiliations:** 1 Division of Endocrinology and Metabolism, Department of Internal Medicine, Chi Mei Medical Center, Tainan, Taiwan; 2 Center of General Education, Chia Nan University of Pharmacy and Science, Tainan, Taiwan; 3 Department of Medical Research, Chi Mei Medical Center, Tainan, Taiwan; 4 Department of Hospital and Health Care Administration, Chia Nan University of Pharmacy and Science, Tainan, Taiwan; Cincinnati Children's Hospital Medical center, United States of America

## Abstract

**Background:**

Obstructive sleep apnea (OSA) is associated with systemic inflammation and induces various comorbid medical diseases. To date, no study has explored the relationship between OSA and atopic dermatitis (AD), an inflammatory and autoimmune skin disorder. This study investigated the longitudinal risk for AD in patients with OSA.

**Methods:**

A random sample of 1,000,000 individuals from Taiwan's National Health Insurance database was collected. From this sample, 1222 patients with newly-diagnosed OSA between 2000 and 2005 were identified and compared with a matched cohort of 18330 patients without OSA. All patients were tracked for 5.5 years from the index date in order to identify which patients subsequently developed AD.

**Results:**

During the 5.5-year follow-up period, the incidence rates of AD in the OSA cohort and comparison groups were 9.81 and 6.21 per 1000 person-years, respectively. After adjustment for age, gender, diabetes, hypertension, coronary heart disease, obesity, allergy, allergic rhinitis, asthma, monthly income, and geographic location, patients with OSA were 1.5-times more likely to develop AD than patients without OSA (95% CI = 1.15–1.95, p = 0.0025). The hazard risk for AD was greater in male OSA patients and young OSA patients (0–18 and 19–34 years), adjusted HRs being 1.53 (95% CI = 1.14–2.06, p = 0.005), 4.01(95% CI = 1.57–10.26, p = 0.0038) and 1.75(95% CI = 1.00–3.04, p = 0.0483), respectively. The log-rank test indicated that OSA patients <35-years-old had significantly higher cumulative incidence rates of AD than those patient of the same age in the comparison group (p = 0.0001).

**Conclusion:**

Patients with OSA, especially male patients and younger patients, are at an increased risk for AD later in life.

## Introduction

Atopy is defined as a condition that involves hyperreactivity to allergic triggers. The prevalence of atopic dermatitis (AD), a chronic inflammatory and pruritic skin disease [1], appears to be increasing, especially in industrialized counties. Although not a life-threatening disease, AD negatively impacts health and quality of life and increases the medical burden. The cause of AD is poorly understood; however, theories include an impaired epidermal barrier, immune system dysregulation, and an inflammatory reaction of the skin [2]. Increasing evidence suggests that AD is more than a local inflammatory disease and may be the result of systemic inflammation. Chemokines, cytokines, T-cells and other inflammatory cells appear to contribute to the initiation and maintenance of this disease [3].

Obstructive sleep apnea (OSA) is a sleep disorder caused by recurrent episodes of upper airway collapse and is characterized by repetitive episodes of apnea/hypopnea and oxygen desaturation during sleep [4]. Recurring arousal from sleep, daytime sleepiness, and fatigue are signs of disturbed sleep. The prevalence of OSA in the general population is approximately 20% [5]. OSA is recognized as a potential cause of many debilitating medical problems, including ischemic heart disease, hypertension, insulin resistance, type 2 diabetes, and mental illness [6]–[8]. Although the underlying pathogenesis linking OSA to these comorbidities is not fully understood, more and more evidence suggests that the systemic inflammation and the procoagulant and thrombotic activity in OSA can lead to endothelial dysfunction, atherosclerosis, and cardiovascular events [9].

OSA has been extensively studied in relation to metabolic and cardiovascular disease (CVD) but rarely in relation to dermatologic disease. Psoriasis, a chronic immune-mediated inflammatory skin disease, is thought to be associated with OSA, metabolic syndrome, and CVD [10]–[12]. The interaction between OSA and psoriasis is very complex and likely linked via systemic inflammation [10], [13], [14]. Few reports have investigated the association between OSA and AD. Some cross-sectional studies have suggested that some OSA features might be associated with atopy, although longitudinal studies are lacking. Chng et al. found atopy to be associated with snoring and other features of OSA [15]. One questionnaire study of children showed an association between snoring and eczema. A case study suggested that continuous positive airway pressure for OSA might resolve dyshidrotic eczema by reducing pro-inflammatory cytokines [16]. Marsell et al. reported that risk factors for snoring were similar to those for allergic disorders [17]. Because both OSA and AD are considered systemic inflammatory diseases, it is reasonable to hypothesize that there might be a relationship between one condition and the development of the other condition. The current study used a national population-based cohort in Taiwan to investigate the risk for the development of AD in patients with OSA over a 5.5-year period.

## Methods

### Data sources

Taiwan launched a single-payer National Health Insurance (NHI) program on March 1, 1995, and as of 2009, the NHI has collected one of the largest and most complete population-based datasets in the world. Data used in this study came from a subset of the NHI database, the Longitudinal Health Insurance Database 2000 (LHID2000), which contains all claims data between 1996 and 2009 from one million beneficiaries randomly selected in 2000 for follow-up study. There are no significant differences in age, gender, and healthcare costs between the sample group and all enrollees. The LHID2000 provides encrypted patient identification numbers, gender, date of birth, dates of admission and discharge, the International Classification of Diseases, Ninth Revision, Clinical Modification (ICD-9-CM) codes of diagnoses and procedures, details of prescriptions, registry of Catastrophic Illness Patient Database, and costs covered by Taiwan's NHI. The institutional review board of Chi Mei Medical Center approved the protocol of this study. The requirement of informed consent was waived because the datasets were devoid of identifiable personal information.

### Study sample

A retrospective cohort study was conducted with two study groups: a new-onset OSA group and a matched non-OSA (comparison) group recruited from 2000-2006. OSA was defined in a patient if (1) he or she had at least two outpatient service claims with the codes of OSA (ICD-9-CM code 78051, 78053, 78057, 32723) at any hospital or local medical clinic and received a polysomnography test or (2) he or she had a single hospitalization for OSA among the 5 claims diagnosis codes. AD was defined in patients if they had been diagnosed on two occasions with AD by dermatologists. Patients diagnosed with OSA before 2000 were excluded, and patients who were diagnosed with AD (ICD-9-CM code 691) before OSA was diagnosed were also excluded. Sex, age, and date of first registration into the NHI program were recorded. Patients not diagnosed with OSA were randomly selected from the dataset and matched with the OSA group by sex, age (±30 days), and index date of first registration into the NHI program and then categorized into the non-OSA group at a ratio of 15 non-OSA for each OSA patient.

For both groups, the geographic area of residence and monthly income (record as NT$) as well as baseline co-morbidities, including diabetes mellitus (ICD-9-CM code: 250), hypertension (ICD-9-CM code: 401-405), coronary heart disease (ICD-9-CM code: 410-414), obesity (ICD-9-CM code: 278), allergy (ICD-9-CM code: 9953, 7080, V150 and V196), allergic rhinitis (ICD-9-CM code: 477) and asthma (ICD-9-CM code: 493) were recorded. These represent critical factors that can affect the risk of AD (ICD-9-CM code: 691). AD might be mediated by oxidative stress and circulating inflammatory cytokines [2], [18]. Since diabetes, hypertension, and coronary heart disease are well-established with increasing inflammatory factors and oxidative stress, these factors were taken in consideration and adjusted for [19], [20]. Obesity was thought to be associated with AD and could represent a trigger for AD [21], [22]. Allergy, allergic rhinitis, and asthma are associated with adaptive immune system dysfunction and are thought to be associated with AD [23]. These comorbidities were included if they presented in an inpatient setting or in three or more ambulatory care claims coded one year before the index medical care date. Person-years (PY) of the follow-up time were calculated for each person until AD diagnosis, death, or the end of the follow-up period in 2009.

### Statistical analyses

All statistical operations were performed using the SAS 9.3 statistical package (SAS Institute, Inc., Cary, North Carolina, USA). Pearson's χ2 tests were used to compare differences in the baseline characteristics, comorbid medical disorders, and sociodemographic status between the two groups. The incidence rate was calculated as the number of AD cases during the follow-up divided by the total person-years for each group by sex and age. Stratified Cox proportional hazard regression, stratified by gender and age group (0–18, 19–34, 35–49, and >50 yr), was performed to calculate the risk for AD between patients with OSA compared to those patients without OSA during the follow-up period. A Kaplan-Meier analysis was used to calculate the cumulative incidence rates for AD between the two cohorts, and the log-rank test was used to analyze the differences between survival curves. A two-sided P value <0.05 was considered significant.

## Results

A summary of the baseline characteristics and comorbid conditions of the two groups is shown in Table 1. A total of 1222 OSA patients and 18330 age-, gender-, and index date-matched non-OSA patients were followed for 5.5 years. Approximately 3% of the group was 0–18 years old, 21% was 19–34 years old, 39% was 35–49 years old, and 37% was ≥50 years old. Eighty percent were males. Patients with OSA had a higher prevalence of diabetes mellitus (DM), hypertension (HTN), coronary heart disease (CHD), obesity, allergy, allergic rhinitis, and asthma. Additionally, patients with OSA had a higher economic status and were more likely to live in central and northern Taiwan than the comparison group.

**Table 1:** Demographic Characteristics and Comorbid Medical Disorders for OSA patients and comparison group patients.

Category	Subcategory	Patients with obstructive sleep apnea (N = 1222)	Comparison patients (N = 18330)	P-value
Age (yr)	0–18	42(3.44%)	630(3.44%)	1.0000
	19–34	258(21.11%)	3882(21.18%)	
	35–49	471(38.54%)	7056(38.49%)	
	≧50	451(36.91%)	6762(36.89%)	
Gender	Female	240(19.64%)	3600(19.64%)	1.0000
	Male	982(80.36%)	14730(80.36%)	
Follow-up years (Mean±SD)		5.50±2.06	5.52±2.05	0.7432
Diabetes	Yes	91(7.45%)	945(5.16%)	0.0005
	No	1131(92.55%)	17385(94.84%)	
Hypertension	Yes	292(23.90%)	1824(9.95%)	<0.0001
	No	930(76.10%)	16506(90.05%)	
Coronary heart disease	Yes	106(8.67%)	498(2.72%)	<0.0001
	No	1116(91.33%)	17832(97.28%)	
Obesity	Yes	11(0.90%)	17(0.09%)	<0.0001
	No	1211(99.10%)	18313(99.91%)	
Allergy	Yes	24(1.96%)	93(0.51%)	<0.0001
	No	1198(98.04%)	18237(99.49%)	
Allergic rhinitis	Yes	94(7.69%)	308(1.68%)	<0.0001
	No	1128(92.31%)	18022(98.32%)	
Asthma	Yes	53(4.34%)	188(1.03%)	<0.0001
	No	1169(95.66%)	18142(98.97%)	
Geographic area	North	751(61.46%)	9247(50.45%)	<0.0001
	Central	277(22.67%)	3206(17.49%)	
	South	191(15.63%)	5472(29.85%)	
	East	3(0.25%)	405(2.21%)	
Income	NT<15840	410(33.55%)	6921(37.76%)	<0.0001
	NT 15841∼25000	268(21.93%)	6008(32.78%)	
	NT>25001	544(44.52%)	5401(29.47%)	

**Figure 1:**
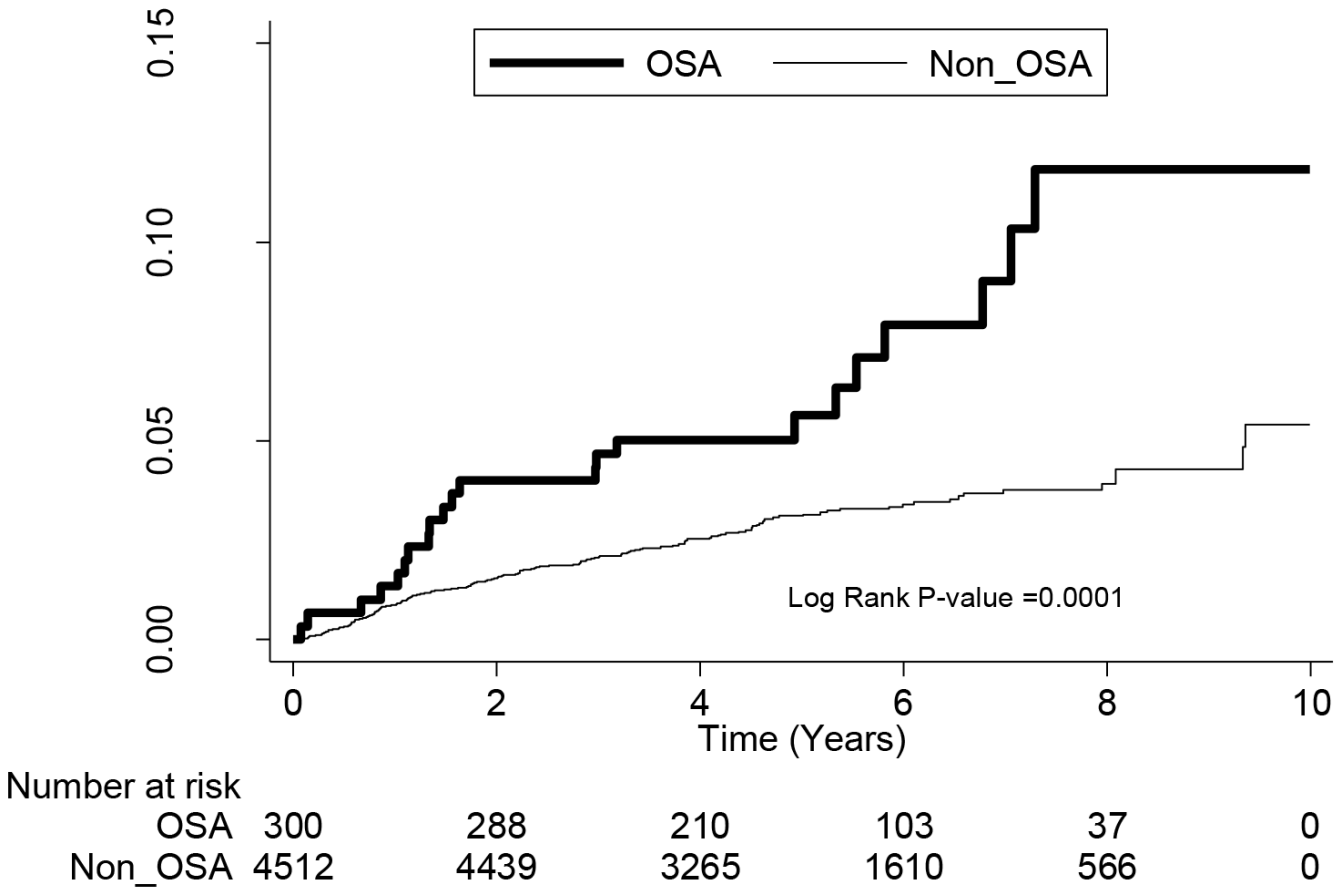
The cumulative incidence rate for atopic dermatitis for patients <35 years of age with obstructive sleep apnea (OSA) and without OSA (log-rank P value  = 0.0001).

**Figure 2:**
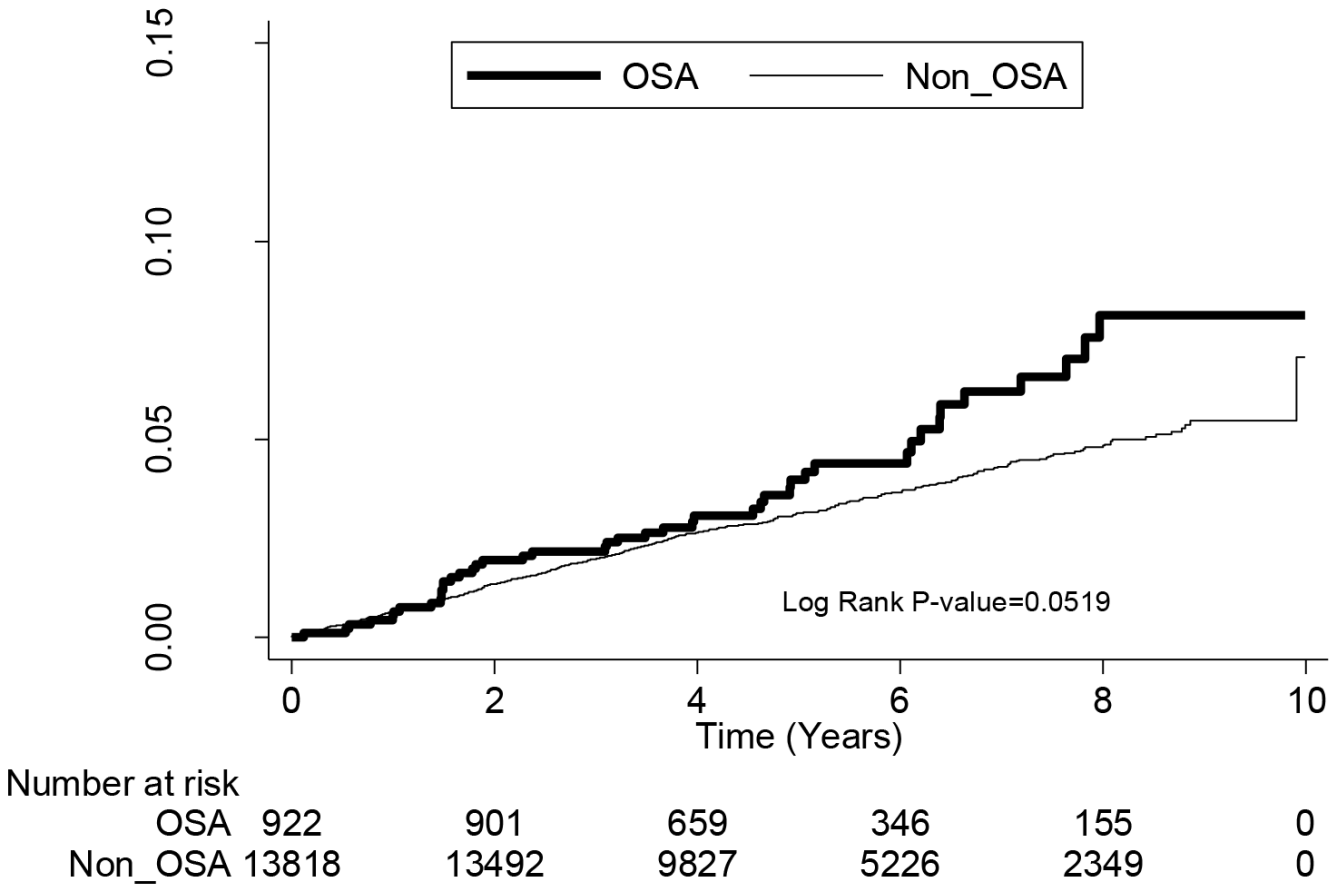
The cumulative incidence rate for atopic dermatitis for patients >35 years of age with obstructive sleep apnea (OSA) and without OSA (log-rank P value  = 0.0519).

Table 2 shows the risk for AD for OSA patients stratified by age and gender. During the 5.5-year follow-up period, the incidence rates for AD for the OSA and non-OSA cohorts were 9.81 and 6.21 per 1000 person-years, respectively. Patients with OSA were at significantly higher risk for AD than those without OSA (crude HR = 1.58, 95% CI = 1.23–2.04, P = 0.0004). After adjusting for age, gender, DM, HTN, CHD, obesity, allergy, allergic rhinitis, asthma, monthly income, and geographic location, OSA patients were 1.5–fold more likely to develop AD (adjusted HR = 1.5, 95% CI = 1.15–1.95, P = 0.0025). The adjusted HRs were most pronounced in the younger age groups 0–18 years and 19–34 years (adjusted HR = 4.01, 95% CI = 1.57–10.26, P = 0.0038 and adjusted HR = 1.75, 95% CI = 1.00–3.04, P = 0.0483, respectively). There were no group differences in risk for AD in the age groups 35–49 years and ≥50 years. Males with OSA were 1.53-times more likely to develop AD than males without OSA after adjusting for confounders (adjusted HR = 1.53, 95% CI = 1.14–2.06, P = 0.0050). No difference in risk for AD was identified among women with and without OSA (adjusted HR = 1.39, 95% CI = 0.79–2.46, P = 0.2522). Kaplan-Meier analysis revealed that OSA patients <35 years of age had a higher cumulative incidence rate for AD than those patients of the same age in the comparison cohort (log-rank test P<0.0001) (Figure 1). In patients >35 years of age, there was no significant difference in cumulative incidence rate for AD between the two groups (log-rank test P = 0.0519) (Figure 2).

**Table 2:** Risk for atopic dermatitis (AD) in patients with and without obstructive sleep apnea (OSA).

		Patients with OSA				Patients without OSA				Crude HR		Adjusted HR	
		N	AD	PY#	Rate*	N	AD	PY#	Rate*	95%CI	P value	95%CI	P value
All		1222	66	6724.62	9.81	18330	629	101232.86	6.21	1.58(1.23–2.04)	0.0004	1.50(1.15–1.95)	0.0025
Age (yr)	0–18	42	6	213.43	28.11	630	26	3473.78	7.48	3.78(1.51–8.93)	0.0041	4.01(1.57–10.26)	0.0038
	19–34	258	16	1394.12	11.48	3882	118	21275.30	5.55	2.07(1.23–3.48)	0.0065	1.75(1.00–3.04)	0.0483
	35–49	471	22	2637.58	8.34	7056	216	39505.95	5.47	1.53(0.98–2.37)	0.0589	1.40(0.88–2.22)	0.1559
	≧50	451	22	2479.49	8.87	6762	269	36977.83	7.27	1.22(0.79–1.88)	0.3716	1.22(0.78–1.90)	0.3927
Gender	Male	982	52	5456.56	9.53	14730	474	81904.02	5.79	1.65(1.24–2.19)	0.0006	1.53(1.14–2.06)	0.0050
	Female	240	14	1268.07	11.04	3600	155	19328.84	8.02	1.38(0.80–2.38)	0.2519	1.39(0.79–2.46)	0.2522

#PY, person-years.

*Rate: per 1000 person-years.

Adjusted by age, gender, diabetes, hypertension, coronary heart disease, obesity, allergy, allergic rhinitis, asthma, monthly income, and geographic location.

## Discussion

Many studies have reported OSA to be comorbid with cardiovascular disease, HTN, type 2 DM, and insulin resistance, but few have investigated the possible relationship between OSA and dermatologic diseases. To the best of our knowledge, this is the first study to investigate the relationship between the OSA and risk for AD. The incidence rate for AD in patients with OSA was 9.81 per 1000 person-years. This study also showed that the incidence for AD was 1.5-times higher in patients with OSA than it was in the age- and gender-matched comparison groups during a 5.5-year follow-up period, after adjusting for age, gender, medical comorbidities, geographic area, and monthly income. The medical comorbidities that were adjusted for have been noted in the literature as risk factors for AD. Although the HR decreased after adjustment, OSA patients still had a significantly higher risk for AD. Subgroup analysis found the risk for developing AD was greater in men with OSA (adjusted HR: 1.53) and younger OSA patients age 0–18years and 19–35 years (adjusted HRs 4.01 and 1.75, respectively).

The prevalence of AD is estimated at 2% to 10% in adults in Europe, where the incidence has grown 2- to 3-fold [24], [25]. One community-based survey in Taiwan by Chen et al. reported the prevalence of AD to be 4.33%, higher than a previous report [26]. Although the prevalence of AD varies from country to country, its incidence appears to be increasing. AD is a very common, pruritic, relapsing inflammatory skin disorder associated with a poor quality of sleep [27]. AD may further aggravate the severity of daytime hypersomnolence in patients with OSA. The wide range of prevalence worldwide seems to suggest expression of this disease may be influenced by racial, environmental, and many other unidentified factors [25]. Although the pathomechanisms underlying AD are not fully understood, the chronic relapsing nature of this disease requires careful monitoring both for the disease itself as well as its associated risk factors. OSA and AD may share similar pathogenic pathways [28]. One study by Al Lawati et al. reported that ethnicity was a risk factor for OSA and that Asians appeared to be at greater risk for OSA at a given body mass index [29]. These findings along with the results of the current study suggest that clinicians in Asia should be aware of the increased risk for AD among patients with OSA.

Although the underlying mechanism contributing to the association between OSA and AD is likely complex, some possible explanations for our findings should be considered. First, obesity is a strong risk factor for OSA, and OSA is a major complication of obesity [30], [31]. According to a study by Hersoug and Linneberg, there is increasing epidemiologic evidence that obesity also increases the risk for AD [21]. This study suggested that obesity might induce decreased immune tolerance and result in atopy [21]. In obese subjects, increases in the circulating levels of interleukin-6 (IL-6) and leptin, and decreases in IL-10 have been reported to down-regulate the immunologic activity of regulatory T-lymphocytes [32]–[35]. Additionally, Vieira et al. reported that obese subjects had a higher frequency of specific IgE positivity than non-obese subjects [36]. These changes could possibly decrease the immunologic tolerance to antigen and possibly result in atopic disease. AD is a disease of altered skin barrier and immune system dysregulation. A defect in filaggrin (FLG) may induce skin barrier dysfunction [37]. One study by Silverberg et al. showed that obesity might act as a trigger in disease associated with FLG mutations and suggested that obesity can be a trigger for AD or exacerbate it [22]. The association between OSA and AD may be in part due to obesity, and this issue merits further investigation. Since obesity was controlled in the present study, the relationship between OSA and AD survived. Obesity alone does not explain the relationship between OSA and AD.

A population-based study by Chung et al investigating the risk factors for AD in Taiwan found DM, CHD, and HTN to be significantly associated with AD (OR = 1.54, P<0.001; OR = 1.36, P<0.001; and OR = 1.22, P<0.001, respectively) [38]. DM increases the expression of pro-inflammatory factors and induces both oxidative stress and endothelial dysfunction [20]. HTN can contribute to oxidative stress, which is an important molecular mechanism associated with cardiovascular disease and tissue injury [19]. One review study by Okayama showed that inflammatory skin diseases like AD are mediated by oxidative stress [18]. Since DM, HTN, and CHD are associated with increasing systemic inflammatory factors and oxidative stress, the association among DM, HTN, and CHD, and AD observed by Chung et al in Taiwan is not surprising [38].Although the true mechanisms contributing to the associations between these comorbidities and AD remain unknown, oxidative stress and systemic inflammation might play substantial roles in this relationship. Therefore, our study examined DM, CHD, and HTN as confounders for AD.

Repeated episodes of hypoxemia during sleep are characteristic of OSA. Periodic hypoxia can lead to activation of sympathetic tone and stimulate the rennin-angiotensin-aldosterone system [39]. Angiotensin II stimulates the release of reactive oxygen species (ROS) and pro-inflammatory cytokines, which initiate and perpetuate oxidative stress and systemic inflammation [40]. The cytokines and chemokines in the circulation and skin cells orchestrate the inflammation reaction in atopic skin [2]. One review study by Okayama showed that inflammatory skin diseases like AD are mediated by oxidative stress [18]. Therefore, one link between OSA and AD might be related to the oxidative stress and systemic inflammation. Furthermore, AD is characterized as a T-helper-_2_ (TH_2_) mediated inflammatory skin disease. The sympathetic activation in OSA increases the release of catecholamine which might induce TH_2_ [41].

A neurocognitive disorder associated with OSA may result from frequent arousals and fragmented sleep. Patients may suffer from psychological problems including daytime sleepiness, poor memory and concentration, irritable mood, anxiety, and emotional stress [29], [42], [43]. Clinical observations have found that psychological stress might act not only as a trigger for but also aggravate the presence of AD [41], [44]. A complete psychological evaluation and specialized care may help alleviate the symptoms of AD and even prevent outbreaks [44], [45].

Age and gender may play a role in modifying the occurrence of AD in patients with OSA. We found a clear relationship between our younger population of OSA patients and increased likelihood of developing AD. Individuals who were diagnosed with OSA later in life were at much less risk for AD. We also found male patients were at higher risk for AD. One prospective clinical epidemiologic study in eastern India showed the prevalence of AD in children to have a slight female-to-male preponderance [46]. Montnemery et al., who conducted a questionnaire study of 12071 adults in Sweden ranging in age from 20 to 59 years [24], found AD to be more frequently reported by women. One previous 8-year national study in Taiwan reported the prevalence of AD to be higher in younger populations and reported that the prevalence decreased with age [47]. That same study reported the prevalence of AD to be lower in females than in males before the age of 8 years, but to increase in females beyond 8 years of age [47]. The underlying reason for the relationship between gender and incidence of AD in OSA patients is unclear. Sex hormones might influence mast cell activation and allergic sensitization. Estradiol promotes sensitization and IgE production, and testosterone suppresses IgE production [48], [49]. AD may, in part, be a consequence of low testosterone in male patients [50]. There is a variable degree of hypogonadism associated with OSA. Male OSA patients may have low testosterone levels, and female OSA patients may have low estradiol levels [31]. Any possible association would be conjecture at this point and would need further investigation.

This study has some limitations. The diagnoses of OSA and AD were based on diagnostic codes recorded by the physician in the NHI claims database; therefore, some registration bias might be involved. To ensure the accuracy of these diagnostic codes, only the OSA patients diagnosed by polysomnography or by two patient claims records citing OSA a primary diagnosis were recorded. The AD patients were those patients who had been diagnosed on 2 occasions with AD by dermatologists. Disease severity could not be assessed from our dataset, and it was not possible to report the association between the severity of OSA and the occurrence of AD. Furthermore, individual information including occupation, mental health, environmental factors, and family history were not available. Further study to investigate the effects of these factors is needed. The strength of this study, however, is that it has a longitudinal, large population-based design. The database covers nearly all of Taiwan's residents, so referral and selection biases were minimized.

In conclusion, we found that patients with OSA are at higher risk for developing AD. The risk was more pronounced in the younger age groups and in men. Further studies are needed to explore the mechanisms underlying these relationships.
